# The Dynamic Changes in the Composition and Diversity of Vaginal Microbiota in Women of Different Pregnancy Periods

**DOI:** 10.3390/microorganisms11112686

**Published:** 2023-11-02

**Authors:** Feifei Hu, Xin Sun, Yao Su, Mingli Huang

**Affiliations:** Department of Obstetrics, The First Affiliated Hospital of Harbin Medical University, Harbin 150001, China; 15045651417@163.com (F.H.); sunxin0932@163.com (X.S.); sy12052023@163.com (Y.S.)

**Keywords:** vaginal microbiota, diversity, structural, pregnant women, *Lactobacillus*

## Abstract

The vaginal microbiota undergoes subtle changes during pregnancy, which may affect different pregnancy responses. This study used the Illumina MiSeq high-throughput sequencing method to analyze the 16S rRNA gene amplicons of pregnant women and the vaginal microbiota structure of pregnant women at different pregnancy periods. There were a total of 15 pregnant women, with 45 samples were taken from these women, within half a year before becoming pregnant, in the last trimester, and 42 days postpartum. Before and after pregnancy, the female vaginal microbiota was mainly composed of *Firmicutes*, followed by *Actinobacteriota* and *Proteobacteria*. The abundance of *Lactobacillus* was relatively high. The α-diversity and microbial abundance were relatively low, and there was no significant difference in microbial composition between the two. After childbirth, the diversity and abundance of women’s vaginal bacterial communities were higher, with a decrease in the number of *Firmicutes* and a higher abundance of *Actinobacteria*, *Proteobacteria*, and *Bacteroidota*. There was a significant difference in the microbial community structure before and after pregnancy. This study showed that the microbiota structure of the vagina of pregnant women was similar to before pregnancy, but after childbirth, there were significant changes in the microbiota of the vagina, with a decrease in the number of probiotics and an increase in the number of harmful bacteria, increasing the risk of illness.

## 1. Introduction

As is well known, the human gut microbiota plays a crucial role in maintaining intestinal homeostasis and preventing disease development. In the past decade, the human gut microbiota has become a widely studied topic. We have a complex microbial community composed of 100 trillion microorganisms in our intestines, which affect human physiology, metabolism, nutrition, and immune function [1,2]. The dynamic changes in the composition and diversity of gut microbiota reflect the health status of the host, and the destruction of intestinal microbiota is related to various gastrointestinal complications, such as Crohn’s disease and ulcerative colitis, as well as complex diseases such as diabetes and obesity [3,4]. Therefore, the human gut microbiota has become a new research hotspot driving the progress of disease diagnosis.

Pregnancy is a special physiological period for women of childbearing age. The vagina has its own microbiota, and changes in vaginal microbiota are related to several pregnancy-related complications. A pregnant woman’s gut microbiome changes significantly to adapt to her changing metabolism and immunity, which plays an important role in maintaining maternal health and fetal development [5]. In recent years, mounting evidence has revealed that gut microbiota dysbiosis is an important internal environmental factor affecting human health, highlighting the significance of the role of gut microbiota during childbirth. During pregnancy, gastrointestinal function and the gut microbiota undergo a series of physiological changes (i.e., metabolic adaptation) to meet the needs of pregnancy [6]. In early pregnancy, gut bacterial products from the intestinal lumen are translocated into the maternal circulation. This is likely associated with increased intestinal permeability and may increase the risk of obstetric complications [7]. Ovarian hormones, including estrogen and progestogen, can change the peripheral and central action mechanism of brain gut axis, regulating the intestinal barrier function and immune activation (IA) of intestinal mucosa. In addition, ovarian hormones can alter the composition of the gut microbiota by affecting bacterial metabolism, growth, and virulence of pathogenic bacteria [4]. Many studies have proved that the changes in gut microbiota during pregnancy are closely related to complications of pregnancy. The normal reproductive tract flora is mainly composed of *Lactobacillus* species bacteria for healthy women, with low bacterial richness and diversity [8]. Research also found that *Lactobacillus* are the main microbiota in the reproductive tract during early pregnancy [9,10], with reduced *Lactobacilli*, increased bacterial diversity, and low vaginal levels of beta-defensin 2 in women with preterm births. In contrast, early and healthy pregnancies are characterized by low diversity and low numbers of bacterial communities dominated by *Lactobacillus*. These observations suggest that early vaginal cultures that show an absence of *Lactobacillus* and polymicrobial vaginal colonization are risk factors for a preterm birth [11]. Once pregnant women become ill, the diversity and abundance of bacteria in the reproductive tract increase. Therefore, it is still necessary to conduct extensive research on the dynamic changes in the gut microbiota of pregnant women during different pregnancy periods, and quantitatively evaluate the changes in bacterial levels that lead to host heterogeneity, especially in the study of a large number of samples. However, in previous reports, researchers have focused on women’s gut microbes, and there has been little research on vaginal flora. Because the vagina can more directly reflect the changes in the microbial community before and after delivery, it is important to study the microbial community structure of different pregnant women.

In this study, we investigated the vaginal microbiota of 15 pregnant women in northern China at the species level, with a wide range of gestational ages, ranging from pre-pregnancy to postpartum. We used 16S rRNA gene sequencing technology and bioinformatics analysis to identify the microbial diversity, composition, and functional characteristics during pregnancy.

## 2. Materials and Methods

### 2.1. Pregnant Women Recruitment and Sample Collection

This study was approved by the Ethics Committee of the First Affiliated Hospital of Harbin Medical University, China (Approval date: 26 December 2022; Reference number: 2022JS35). Written informed consent from all eligible participants was obtained, in accordance with the principles of the Declaration of Helsinki. The pregnant women were recruited from the First Affiliated Hospital of Harbin Medical University from January 2022 to March 2023. There were a total of 15 pregnant women, with 45 samples were taken from these women, within half a year before becoming pregnant, in the last trimester, and 42 days postpartum. All pregnant women were healthy women of childbearing age, aged 20–40, who were preparing to conceive and had no medical or gynecological diseases or inflammation before pregnancy. The pregnancy methods were all natural conception, and there was no use of hormones or artificial intervention during pregnancy to assist with pregnancy. Newborns were delivered through natural vaginal delivery. The pregnant women took oral vitamins and appropriate calcium supplements before and during pregnancy and underwent regular prenatal examinations after pregnancy. When sampling, women were required to not use antibiotics and antifungal drugs within 30 days, to not engage in sexual activity within 48 h before sampling and to not wash the vulva or apply medicine. During sampling, the vulva or urethral opening was disinfected with conventional aseptic procedures, the vagina was dilated with a speculum, and secretions from the cervix opening or posterior cervical dome were swabbed with a sterile sponge swab, without touching the vaginal wall, and rolled in a circle five times before being placed into a sterile swab tube. These samples were immediately stored at −80 °C until DNA extraction.

### 2.2. DNA Extraction and 16S rDNA Amplicon Sequencing

DNA from vaginal secretion samples was extracted using an Omega M5635–02 Kit (Omega Bio Tek, Norcross, GA, USA), according to the manufacturer’s instructions. All experiments were carried out on a super-clean table. The concentration and purity of the DNA was quantified by a NanoDrop 2000 spectrophotometer (Thermo Fisher Scientific, Wilmington, DE, USA). The V3–V4 hypervariable regions of the bacterial 16S rDNA were amplified by a 2-step PCR method forward primer (5-CCTACGGGNGGCWGCAG-3) and the reverse primer (5-GACTACHVGGGTATCTAATCC-3), with unique 8 bp barcodes to facilitate multiplexing. Sequencing was performed with an Illumina PE250 platform at Majorbio Bio-Pharm Technology Co., Ltd. (Shanghai, China).

### 2.3. Bioinformatics and Statistical Analysis

The paired-end (PE) reads obtained from the Illumina PE250 sequencing were first spliced according to the overlap relationship, and, at the same time, the sequence quality was controlled and filtered. After distinguishing samples, the remaining clean readings were classified as operational taxon (OTU) with a 97% sequence similarity level cluster. All OTUs were then annotated based on the ribosome database project (RDP) and readings for each spike in the OTU were calculated. Based on the OTU cluster analysis results, the OTU can be analyzed for a variety of diversity indexes, and the sequencing depth can be detected [12]. The relative abundance of each bacterium, from phylum to genus level, was measured using the QIIME pipeline [13]. Chao1, Shannon, and Simpson indices were calculated to evaluate α-diversity within the group. β-Diversity was assessed by principal components analysis (PCA) and principal coordinate analysis (PCoA) of the unweighted UniFrac (unique fraction) distance matrix and was visualized using a non-metric multidimensional scaling (NMDS) graph [14]. The abundance results of each sample’s taxonomic group at the phylum and genus levels were compared with the transfer and were displayed in a histogram. Using the gplots R package, the bacteria with the largest relative abundance at the level of the amplification sequence variant (ASV) were mapped as heat maps. The linear discriminant analysis effect size (LEfSe) tool was used to identify the taxa that may show significant differences in the group.

### 2.4. Statistical Analysis

Statistical analyses were performed using R software (version 3.6.1). Continuous variables were reported as means ± standard deviations. Student’s *t*-tests were used to study differences in continuous variables. A *p*-value of < 0.05 was considered statistically significant.

## 3. Results and Discussion

### 3.1. OTU Level Analysis

**Figure 1:**
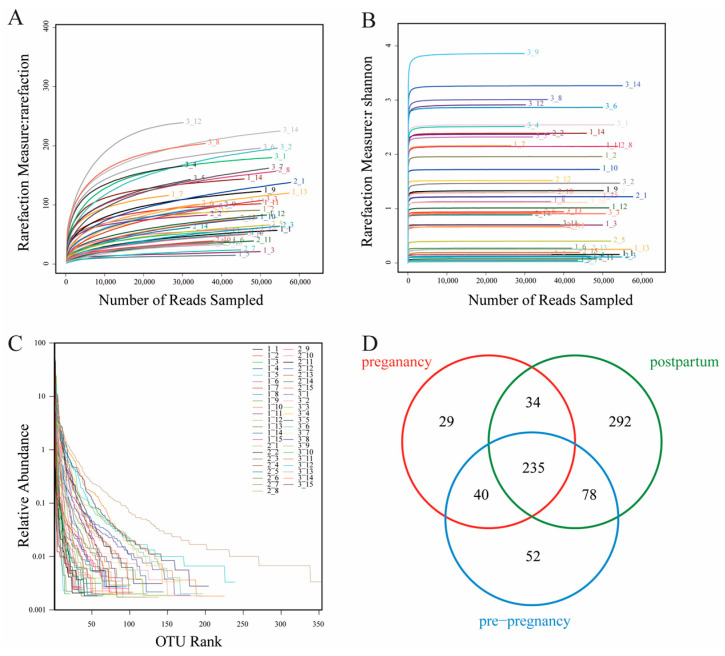
Rarefaction curve (**A**), Shannon–Wiener index (**B**), rank abundance (**C**), and Venn diagram of the overlap at the OTU level (**D**) in different groups.

**Figure 2:**
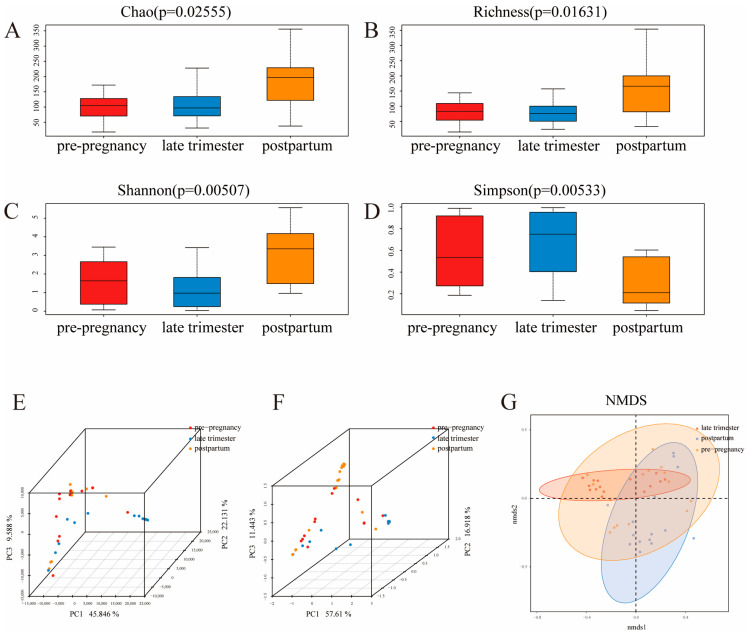
α and β diversity index in different samples. (**A**) Chao1 index; (**B**) richness; (**C**) Shannon index; (**D**) Simpson index; (**E**) PCA score plot; (**F**) PCoA score plot; (**G**) NMDS. Data are expressed as the mean ± SD (*n* = 15). Data with different letters indicate a significant difference (*p* < 0.05).

The microbial composition in pre-pregnancy, pregnancy, and postpartum was measured by 16S rRNA sequencing. A total of 411 16S rDNA raw gene sequences were obtained from 45 samples through Illumina Miseq sequencing. The average number of sequences from each sample was 2,075,315. The different pregnancy periods influenced more OTUs of vaginal bacteria and they also showed stronger effects on vaginal microbial taxonomy. The results of the curve of rarefaction and the Shannon–Wiener index indicated that the deep sequencing covered most of the OTUs, and the sequencing data were large enough to reflect the vast majority of microbial information in the sample (Figure 1A,B). The rank abundance curves reflected the uniform distribution of species distribution in the sample (Figure 1C). As the Venn diagram showed (Figure 2A), a total of 760 OTUs were discovered after sequencing, in which 52, 29, and 292 OTUs were specifically found in pre-pregnancy, pregnancy, and postpartum women, respectively.

### 3.2. α and β Diversity Analysis

The Chao1 and richness indices are often used to estimate the abundance of microbial communities in the sample, and the larger the value, the richer the abundance in the community [15]. The Shannon index is often used to evaluate the microbial diversity in the sample, and the larger the value, the higher the community diversity. Simpson is a commonly used indicator for evaluating uniformity [16]. The α diversity results are shown in Figure 2. According to the results of the Chao1 and richness indices, the total microbial species diversity reached the highest levels in the postpartum group, which was significantly higher than that of the pre-pregnancy and pregnancy groups (*p* < 0.05) (Figure 2A,B). Compared with different samples, the Simpson and Shannon indices also confirmed that there were varying degrees of differences in the uniformity and diversity of the microbiota, indicating that pregnancy time significantly affects the diversity of the vaginal microbiota (Figure 2C,D). The abundance of microbial communities showed an increased trend postpartum, but there was no significant difference in community diversity between pre-pregnancy and pregnancy groups. β diversity analyses including PCA and PCoA were performed to visualize the differences in microbiota structure among the three groups [17]. The unweighted UniFrac distance was calculated to reflect the difference in community composition between groups (Figure 2E–G). The clustered points in each group showed a well parallelism, and the distinct distance between groups reflected the variation in microbial communities. The results indicated an obvious clustering of the vaginal microbial communities among different samples (Figure 2E: PC1, PC2, and PC3 were 45.846%, 22.131%, and 11.588%, respectively; Figure 2F: PC1, PC2, and PC3 were 57.610%, 16.918%, and 11.443%, respectively). Vaginal microbiota in different pregnancy stages exhibited distinctive microbiota profiles. The pre-pregnancy and postpartum groups exhibited a visualized separation, certifying that the pregnancy period had a material impact on the vaginal microbial composition of pregnant women.

### 3.3. Changes in Community Structure and Composition

**Figure 3:**
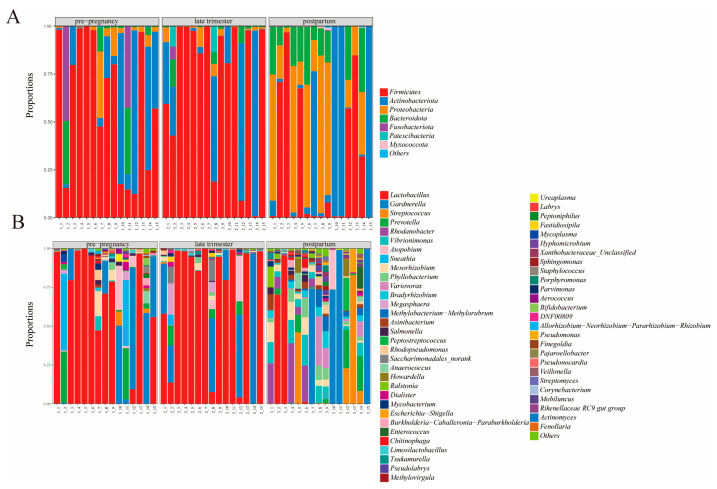
Vaginal microbiota composition among experimental groups at the phylum (**A**) and genus (**B**) level.

Figure 3A analyzed the microbiota community composition of different groups at the phylum level. A total of seven types of bacteria were detected in different samples, including *Firmicutes*, *Actinobacteriota*, *Proteobacteria*, *Bacteroidota*, *Fusobacteriota*, *Patescibacteria*, and *Myxococcota*. This result was consistent with the research of Xiao et al., who found that *Firmicutes*, *Actinobacteriota*, *Proteobacteria*, *Bacteroidota*, and *Fusobacteriota* were also the main microbiota of human colonic microbiota [18]. *Firmicutes* and *Actinobacteriota* belong to two dominant groups, accounting for about 90–99%, followed by *Proteobacteria*. The relative abundance of four vaginal microbiotas changed significantly: *Firmicutes*, *Actinobacteriota*, *Proteobacteria*, and *Bacteroidota*. *Patescibacteria* and *Myxococcota* only exist in individual samples in the pregnancy group and postpartum group, respectively. We found that the relative abundance of the phylum *Firmicutes* was significantly higher in the pre-pregnancy and pregnancy groups than that of the postpartum group (*p* < 0.05), while that of *Proteobacteria* was enriched, which was in keeping with the results of previous studies in pregnant women [19]. *Bacteroidota*, a type of Gram-negative bacteria, is the main contributor to lipopolysaccharides biosynthesis. Therefore, high abundances of *Bacteroidetes* may induce increased inflammation during pregnancy [20]. Compared with the pre-pregnancy and pregnancy groups, the relative abundance of *Bacteroidota* in postpartum group significantly increased. The ratio of *Firmicutes/Bacteroidetes* is related to obesity and diabetes and is considered as a health index of the intestinal microbiota. Research showed that the abundance of *Firmicutes* in obese patients was higher, while the abundance of *Bacteroidetes* was significantly lower than that in healthy people. Liu et al. also found that the ratio of *Firmicutes/Bacteroidetes* in the gut microbiota of healthy people was high [21]. Previous studies have shown that the increase in *Bacteroidetes* was associated with overweight and obesity in pregnant women, which may increase the risk of intrauterine diseases in pregnant women [22,23]. *Actinobacteria* are Gram-positive bacteria, which are one of the probiotics in the intestinal tract beneficial to human health [24]. They have a variety of physiological functions such as enhancing intestinal immunity, anti-tumor properties, and the influence of probiotics on gastrointestinal function [25,26]. Some studies have shown that *Proteobacteria*, using flagella movement, is a mucinous bacterium that can aggregate to form a multicellular matrix, and excessive amounts can lead to mucositis [27]. Additionally, *Fusobacteria* mainly appear in the pre-pregnancy group, while *Patescibacteria* mainly appear in the pregnancy group [28]. Therefore, the number of probiotics in the vagina of pregnant women decreases, the physical condition becomes weaker, and the risk of disease increases after childbirth.

The genus-level sequences *Lactobacillus*, *Gardnerella*, *Streptococcus*, *Prevotella*, *Rhodanobacter*, and *Vibrionimonas* were the most abundant bacteria (Figure 3B). The relative abundance of *Lactobacillus* was significantly different. Compared with the pre-pregnancy group, the pregnancy group’s relative abundance of *Prevotella* and *Sneathia* reduced significantly (*p* < 0.05). The core microbiota was still *Lactobacillus*, but in some samples, *Gardnerella* was the core microbiota. The diversity of the microbiota of the pre-pregnancy group was higher than that of the pregnancy group (*p* < 0.05), while *Lactobacillus* were no longer the core of the microbiota and the diversity of the microbiota significantly increased, in the pregnancy group. The abundance of *Lactobacillus* was significantly decreased in the postpartum group compared to the pre-pregnancy group and the pregnancy group (*p* < 0.05). The postpartum group had significantly increased abundances of *Streptococcus*, *Prevotella*, *Rhodanobacter*, and *Vibrionimonas* (*p* < 0.05). In individual samples, *Streptococcus* and *Prevotella* became the core microbiota. Previous studies have shown that *Lactobacillus* contains abundant probiotics, which can effectively inhibit the growth of pathogenic bacteria, such as *Helicobacter pylori*, and promote human health [18]. Intestinal probiotics can improve the host’s intestinal microenvironment, helping the body improve its intestinal and immune functions. Probiotics can control pregnancy weight gain and reduce complications of pregnancy by inducing changes in the intestinal microbiome during pregnancy [29]. Nordqvist et al. [30] found that the consumption of specific probiotics by pregnant women can significantly reduce the incidence of premature birth in early pregnancy and significantly reduce the incidence of PE in late pregnancy. Studies have shown that, compared with normal pregnant women, pregnant women with gestational diabetes have an imbalance of the intestinal flora, a decrease in the number of *Lactobacillus* and *Bacteroides* in the gut, and an increase in the number of intestinal bacteria [31]. *Escherichia-Shigella* prefers to grow in weakly alkaline environments [32]. Our study found that the number of *Escherichia-Shigella* in individual samples of the postpartum group increased, possibly due to changes in the acid–base environment of the pregnant woman’s intestine after delivery. Some studies have shown that *Streptococcus* is involved in carbohydrate metabolism and related to various metabolic disorders [33]. This study found that increasing the abundance of *Streptococcus* in individual samples after delivery may affect the physical health of pregnant women. *Lactobacillus* can maintain the stability of the uterine environment in pregnant women. High estrogen can induce *Lactobacillus* to break down glycogen and lactate. Low uterine pH is also beneficial for the production of *Lactobacillus* and protection from harmful bacteria [34]. Therefore, the pH value of the vagina can be used as a predictive indicator of uterine infection. The reduction in *Lactobacillus* in the vagina led to an increase in the richness and diversity of bacterial genera, and the vagina was colonized by potential pathogens, including the *Prevotella*, *Megasphaera*, *Anaerococcus*, and *Peptoniphilus* genera [35,36]. The study found that the reduction in *Lactobacillus* was associated with poor reproductive health. In the results of this study, *Lactobacillus* was also the main sample in the vagina, followed by *Gardnerella*, but, postpartum, *Lactobacillus* was significantly reduced. Moreover, various genera of the phylum *Mycoplasmatota* are considered part of the vaginal microbiome and opportunistic pathogens. Rumyantseva et al. [37] estimated the prevalence of *Mycoplasma hominis*, *Ureaplasma parvum*, and *Ureaplasma urealyticum* in healthy women and patients with altered vaginal microflora. They found that *Mycoplasmas* was a marker or a symbiont of the vaginosis flora. Therefore, we can further explore the changes in *Mycoplasma* in the vaginas of women during different pregnancy periods, and further analyze the microbial community structures in the vaginas of pregnant women in the future study.

**Figure 4:**
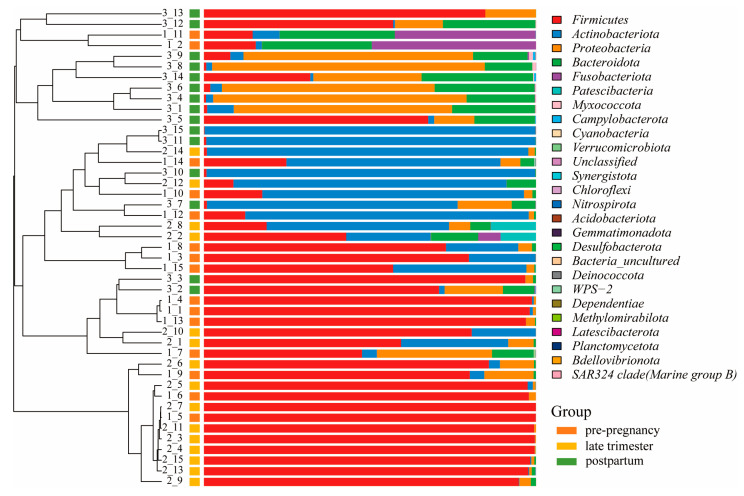
Microbial community bar plot with cluster tree.

The hierarchical clustering tree is based on the Bray–Curtis method, which reflects the degree of difference in different samples in the distance between the points. The results showed a separation between the pre-pregnancy, pregnancy, and postpartum groups, which revealed that the vaginal microbial composition varies considerably in response to different delivery periods (Figure 4). The distribution of predominant bacteria at the phylum level in the pre-pregnancy group was similar to that of the pregnancy group and significantly separated from the postpartum group, from which the pregnancy group was partially separated. The pre-pregnancy group and the pregnancy group clustered into one category in the clustering tree, and then clustered with the postpartum group, indicating that the pre-pregnancy group was closer to the pregnancy group’s species distribution. *Firmicutes* serve as the core microbiota of the pre-pregnancy group and the pregnancy group. With the extension of pregnancy, *Actinobacteriota* became the core microbiota in some pregnancy group samples. After delivery, the number of *Firmicutes* sharply decreased, while the number of *Proteobacteria* and *Bacteroidota* increased, which was significantly different from the pre-pregnancy and pregnancy groups. Consistent with the results, the hierarchical clustering tree implied distinct microbiota profiles among the pre-pregnancy, pregnancy, and postpartum groups, with the pregnancy group being more similar to the pre-pregnancy group. Previous studies have found that chronic inflammation is associated with an increased proportion of *Firmicutes* and *Bacteroidota* [38]. Cytokines are produced by inflammatory reactions, and cytokine proteins protect the body from infection and interfere with iron processing and red blood cell production [39]. Therefore, these data demonstrate that childbirth leads to an increase in the diversity of the vaginal flora of pregnant woman and a decrease in the content of *Firmicutes*.

**Figure 5:**
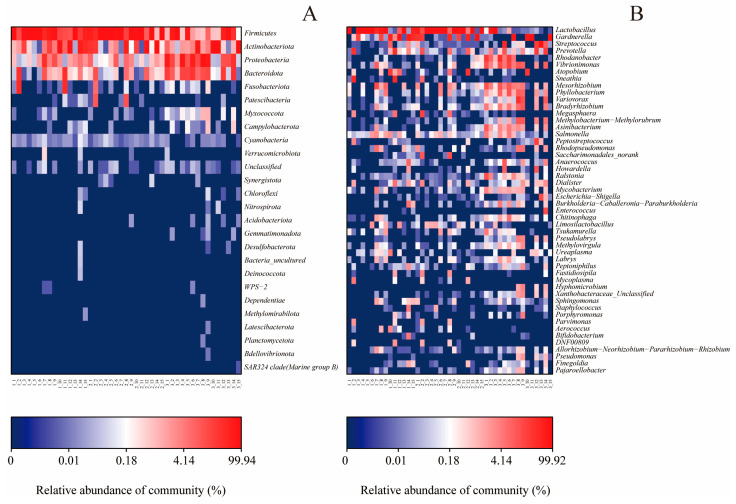
Heat-map showing the relative abundance of key genera at the phylum (**A**) and genus (**B**) level of different treatment groups.

In addition, a heatmap analysis of the relative abundances of 26 genera was performed, to evaluate the distribution of gut microbes in each group (Figure 5). At the phylum level, *Firmicutes*, *Actinobacteriota*, *Proteobacteria*, and *Bacteroidota* were the dominant taxa in all groups. Furthermore, the abundance of *Firmicutes* was significantly higher in both the pre-pregnancy and pregnancy groups, compared to the postpartum group (*p* < 0.05) (Figure 5A). The relative abundance of *Actinobacteriota*, *Proteobacteria*, and *Bacteroidota* in the postpartum group was higher than those in the pre-pregnancy group and the pregnancy group (*p* < 0.05). The relative abundance of *Patescibacteria* in the pregnancy group was lower than that in the pre-pregnancy group and the pregnancy group (*p* < 0.05). At the genus level, compared with the postpartum group, *Lactobacillus* was significantly increased (*p* < 0.05) in abundance in the pre-pregnancy and pregnancy groups, whereas *Streptococcus*, *Prevotella*, *Rhodanobacter*, and *Vibrionimonas* were significantly decreased (*p* < 0.05) in abundance in the microbiota (Figure 5B). In the postpartum group, the relative abundances of *Mesorhizobium*, *Phyllobacterium*, *Variovorax*, *Bradyrhizobium*, and *Methylobacterium* were significantly increased, compared with the other groups (*p* < 0.05). Moreover, delivery increased the relative abundance of *Salmonella*, *Anaerococcus*, *Ralstonia*, *Dialister*, *Mycobacterium*, and *Escherichia-Shigella* and decreased the relative abundance of *Saccharimonadales* and *Megasphaera*, compared with the pre-pregnancy and pregnancy groups.

### 3.4. Community Structure and LEfSe Variance Analysis

**Figure 6:**
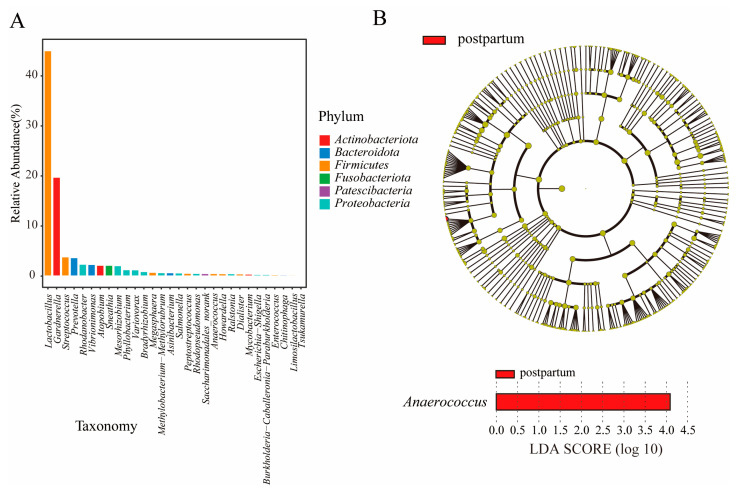
Component diagram of dominant species community structure (**A**) and dominant species bar plot and plot LEfSe (**B**) in postpartum group.

The analysis of the community structure of dominant species indicated that *Firmicutes* were the main components of the vaginal microbiota of pregnant women, followed by *Actinobacteriota*, *Bacteroidota*, *Proteobacteria*, *Fusobacteriota*, and *Patescibacteria*. *Firmicutes* mainly include *Lactobacillus*, *Streptococcus*, *Megasphaera*, *Peptostreptococcus*, *Anaerococcus*, *Howardella*, *Dialister*, *Enterococcus*, and *Limosilactobacillus* (Figure 6A). Some studies found that many factors may affect the stability of the uterine microbiota, including different types of *Lactobacillus*. Yang et al. [40] studied the gut microbiota of pregnant women and its relationship with host factors and found that individual heterogeneity is the main factor affecting the gut microbiota during pregnancy. Our findings were consistent with those of Yang et al., as the vaginal microbiota was associated with the different stages of pregnancy. *Actinobacteriota* mainly includes *Gardnerella*, *Atopobium*, *Mycobacterium*, and *Tsukamurella*. *Bacteroidota* mainly include *Prevotella*, *Asinibacterium*, and *Chitinophaga*. To further investigate the dominant bacteria of the postpartum group, LEfSe analysis (LDA effect size) was performed to identify the specific predominant bacterial phenotypes. Fifty-one discriminatory genera were identified as key discriminants (Figure 6B). The results indicated that the genera *Anaerococcus* was significantly enriched in the postpartum group (*p* < 0.05).

The results showed that the vaginal microbiota affects or reflects the regulation of the duration of gestation and labor onset, with potentially vast clinical utilities. The vaginal microbiota is, at least conceptually, modifiable, and our findings highlight the importance of future studies to understand the nature of implicated host–microbiota interactions. In future research on vaginal microbiota, we need to increase the sample size of pregnant women and address the causality and mechanisms regarding how pregnancy affects the vaginal microbiota. Moreover, whether the microbiota changes just reflect or actively contribute to the underlying immunological processes and mechanisms remains to be studied, and its potential for diagnostic and therapeutic approaches remains to be investigated.

## 4. Conclusions

The human microbiota plays a central role in health and female morbidity. Therefore, classifying women based on bacterial patterns would allow for a personalized microbiota-based diagnosis, which could then be used to develop personalized therapies for disease prevention and personalized treatments. We evaluated the microbial community structure of the reproductive tract of pregnant women at different stages of pregnancy through 16S rRNA sequencing. In this study, we found significant changes in the diversity and composition of microbial communities at different stages of pregnancy. There was no significant difference in the microbiota of the vagina between pre-pregnancy and post-pregnancy women, with the main microbiota being *Lactobacillus*. After childbirth, the diversity of bacterial communities in the female vaginal decreases, the number of *Lactobacillus* decreases, and the number of pathogenic bacteria, such as the *Prevotella*, *Megasphaera*, *Anaerococcus*, and *Peptoniphilus* genera, increases. This indicates that the vaginal microbiota of women after childbirth is not coordinated and carries a risk of illness.

## Data Availability

The data presented in the study are deposited in the National Center for Biotechnology Information (NCBI), accession number SRP456518.
